# Genome Sequence of Arthrobacter globiformis Phage KeAlii from Hawaiʻi

**DOI:** 10.1128/mra.00439-22

**Published:** 2022-07-20

**Authors:** Rebecca A. Chong, University of Hawaiʻi at Mānoa SEA-PHAGES students, Stuart P. Donachie, Floyd A. Reed, Megan L. Porter

**Affiliations:** a School of Life Sciences, University of Hawaii at Mānoa, Honolulu, Hawaiʻi, USA; Queens College CUNY

## Abstract

Here, we report the genome sequence of bacteriophage KeAlii, a *Siphoviridae* that infects Arthrobacter globiformis strain B-2979, from Honolulu, Hawaiʻi. The 41,850-bp genome contains 66 predicted protein-coding genes and 1 gene that encodes a tRNA for tryptophan. Genome comparisons suggest KeAlii is closely related to actinobacteriophage Adolin.

## ANNOUNCEMENT

Understanding the molecular evolution of bacteriophages is critical to finding potential medical solutions for antibiotic resistance in bacteria as well as diverse applications in agricultural and biotechnological settings. Here, we present the genome sequence of the actinobacteriophage KeAlii that infects the soil bacterium Arthrobacter globiformis strain (str.) B-2979^T^, providing one of the first sequenced actinobacteriophage genomes from Hawaiʻi through the SEA-PHAGES program (1, 2).

**FIG 1:**
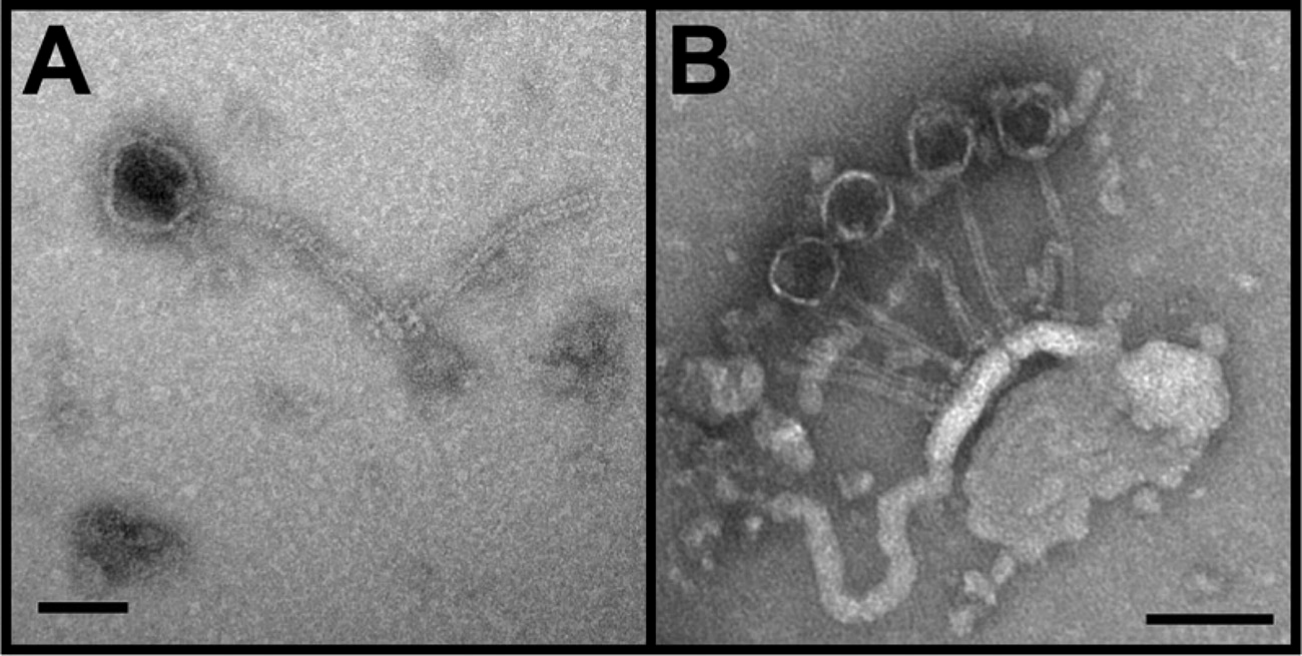
Transmission electron microscopy imaging of KeAlii reveals an icosahedral head (A) and a noncontractile tail (B) that are characteristic of *Siphoviridae* phages. A high-titer (>1.0 × 10^5^ PFU/mL) lysate was negatively stained with 1% uranyl acetate. The scale bars correspond to 50 nm in A and 100 nm in B.

KeAlii was isolated and purified in 2020 from a surface soil sample collected at the University of Hawaiʻi at Mānoa (UHM) in Honolulu, on Oahu, Hawaii (21.3265 N, 157.8024 W), on a sunny day with an ambient temperature of 30°C. Using a direct isolation method as outlined in the SEA-PHAGES manual (3), KeAlii was isolated on peptone yeast calcium agar (PYCa) medium incubated at 22°C with the host *A. globiformis* str. B-2979^T^. Imaging of KeAlii by transmission electron microscopy revealed an icosahedral head and a noncontractile tail that are characteristic of *Siphoviridae* phages (Fig. 1).

Phage particles were confirmed and purified via plaque assay and then amplified to a high titer for genomic DNA extraction for sequencing. Total genomic DNA extractions were performed at UHM using a Wizard DNA extraction kit (Promega) following the manufacturer’s protocols. A sequencing library was prepared at the Pittsburgh Bacteriophage Institute with an NEBNext Ultra II FS DNA Library Prep Kit with dual-indexed barcoding, and sequencing was completed using the Illumina MiSeq platform resulting in 280,361 single-end 150-bp reads. Default parameters were used for all software unless otherwise specified. Raw reads were assembled in Newbler v2.9 (Roche), resulting in a single genomic contig with 922-fold coverage. Genome completeness, accuracy, and phage genomic termini were verified using Consed29 (4–6). The complete genome sequence of KeAlii (GenBank accession OK040777.1; assembly ASM2068489v1) is 41,850 bp in size with a G+C content of 65.5% and has a characteristic physical end with an 11-bp 3′ sticky overhang.

The genome of KeAlii was annotated using Glimmer v3 (7) and GeneMark v2 (8); resulting automated annotations were verified manually using DNA Master v5.23.6 build 2701 (http://cobamide2.bio.pitt.edu/computer.htm), Phage Evidence Collection and Annotation Network (PECAAN; http://pecaan.kbrinsgd.org), Phamerator (9), and Starterator (http://phages.wustl.edu/starterator/). KeAlii is predicted to have 67 genes, as follows: 1 tRNA gene, 33 genes (50%) have a putative function assigned, and the remaining 33 genes (49%) encode hypothetical proteins with unknown functions. Potential functions for predicted protein-coding genes were assigned based on top hits for searches using NCBI BLASTP (10) and HHpred (11), and putative membrane proteins were identified using TMHMM v2.0 (now DeepTHMHMM https://services.healthtech.dtu.dk/service.php?DeepTMHMM).

Actinobacteriophages sharing at least 50% nucleotide identity are arranged into clusters, with KeAlii falling in the AZ cluster of actinobacteriophages. KeAlii is predicted to be a temperate phage, as a serine integrase was identified (gene 47), although no immunity repressor gene was identified. Other interesting genes identified include a VIP2-like ADP-ribosyltransferase toxin gene (gene 4) and a putative endolysin (gene 24). KeAlii is most genetically similar to Adolin (GenBank accession MN813676.1), with 81.67% nucleotide identity via BLAST alignment.

### Data availability.

The complete genome sequence of actinobacteriophage KeAlii has been deposited in GenBank with accession number OK040777, BioProject accession number PRJNA488469, and SRA accession number SRX15105353.
